# Crystal structure of bis­(9*H*-6-amino­purin-1-ium) hexa­fluorido­silicate(IV) dihydrate

**DOI:** 10.1107/S2056989014027005

**Published:** 2015-01-03

**Authors:** Ratiba Belhouas, Sofiane Bouacida, Chaouki Boudaren, Jean-Claude Daran, El Hossain Chtoun

**Affiliations:** a Unité de Recherche de Chimie de l’Environnement et Moléculaire Structurale, CHEMS, Université Mentouri-Constantine, 25000, Algeria; b Département Sciences de la Matière, Faculté des Sciences Exactes et Sciences de la Nature et de la Vie, Université Oum El Bouaghi, Algeria; c Laboratoire de Chimie de Coordination, UPR CNRS 8241, 205 route de Narbonne, 31077 Toulouse Cedex, France; d Université Abdelmalek Essaadi, Faculté des Sciences, BP 2121 M’Hannech II, 93002 Tétouan, Morocco

**Keywords:** crystal structure, purinium cation, hexa­fluorido­silicate anion, hydrogen bonding

## Abstract

The asymmetric unit of the title compound, 2C_5_H_6_N_5_^+^·SiF_6_^2−^·2H_2_O, contains one adeninium cation, half of a hexa­fluorido­silicate anion located on an inversion centre and one lattice water mol­ecule. The adeninium cations are connected through N—H⋯N hydrogen bonds involving one H atom of the –NH_2_ group and the H atom of the protonated N atom of the adenine ring system, forming centrosymmetric ring motifs of the type *R*_2_^2^(10) and *R*_2_^2^(8), respectively. The overall connection of the cation leads to the formation of planar ribbons parallel to (122). In the ribbons, slipped π–π stacking inter­actions, with a centroid-to-centroid distance of 3.6938 (9) Å, an inter­planar distance of 3.455 Å and a slippage of 1.306 Å is observed. The hexa­fluorido­silicate anion and the water mol­ecule are linked through O—H⋯F hydrogen bonds [ring motif *R*_4_^4^(12)] into chains parallel to [100]. The cationic ribbons and anionic chains are finally connected through additional N—H⋯O, N—H⋯F and O—H⋯F hydrogen bonds into a three-dimensional network in which layers of adeninium cations and fluorido­silicate anions alternate parallel to (001).

## Related literature   

The title compound was prepared as part of our ongoing studies of hydrogen-bonding inter­actions in the crystal structures of protonated amines (Bouacida *et al.*, 2005*a* ▸,*b* ▸,*c* ▸; 2006 ▸; Belhouas *et al.*, 2012 ▸). For π–π stacking inter­actions, see: Janiak (2000 ▸).
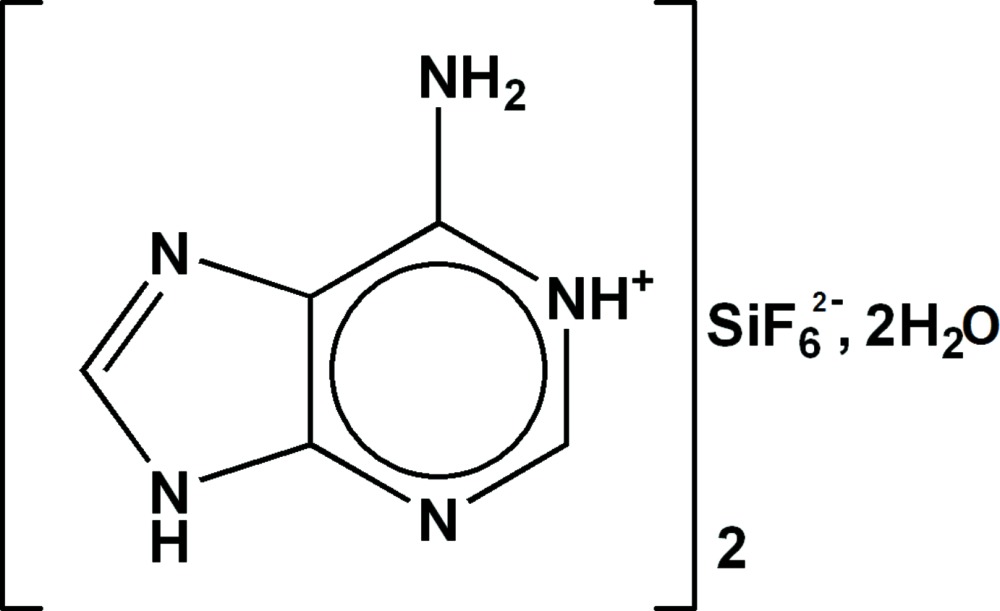


## Experimental   

### Crystal data  

2C_5_H_6_N_5_^+^·SiF_6_^2−^·2H_2_O
*M* *_r_* = 450.42
Triclinic, 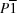

*a* = 5.7500 (7) Å
*b* = 7.8504 (3) Å
*c* = 10.0884 (6) Å
α = 79.141 (6)°
β = 84.534 (17)°
γ = 71.774 (9)°
*V* = 424.47 (6) Å^3^
*Z* = 1
Mo *K*α radiation
μ = 0.24 mm^−1^
*T* = 295 K
0.55 × 0.12 × 0.07 mm

### Data collection  

Nonius KappaCCD diffractometer
4025 measured reflections
1923 independent reflections
1757 reflections with *I* > 2σ(*I*)
*R*_int_ = 0.018

### Refinement  

*R*[*F* ^2^ > 2σ(*F* ^2^)] = 0.030
*wR*(*F* ^2^) = 0.082
*S* = 1.03
1923 reflections
133 parameters
H-atom parameters constrained
Δρ_max_ = 0.28 e Å^−3^
Δρ_min_ = −0.22 e Å^−3^

### 

Data collection: *COLLECT* (Otwinowski & Minor, 1997 ▸); cell refinement: *DIRAX/LSQ* (Duisenberg *et al.*, 2003 ▸); data reduction: *EVALCCD* (Duisenberg *et al.*, 2003 ▸); program(s) used to solve structure: *SIR92* (Burla *et al.*, 2005 ▸); program(s) used to refine structure: *SHELXL97* (Sheldrick, 2008 ▸); molecular graphics: *ORTEPIII* (Burnett & Johnson, 1996 ▸), *ORTEP-3 for Windows* (Farrugia, 2012 ▸) and *PLATON* (Spek, 2009 ▸); software used to prepare material for publication: *WinGX* (Farrugia, 2012 ▸).

## Supplementary Material

Crystal structure: contains datablock(s) SiAde, I. DOI: 10.1107/S2056989014027005/wm5099sup1.cif

**Fig. 1.**
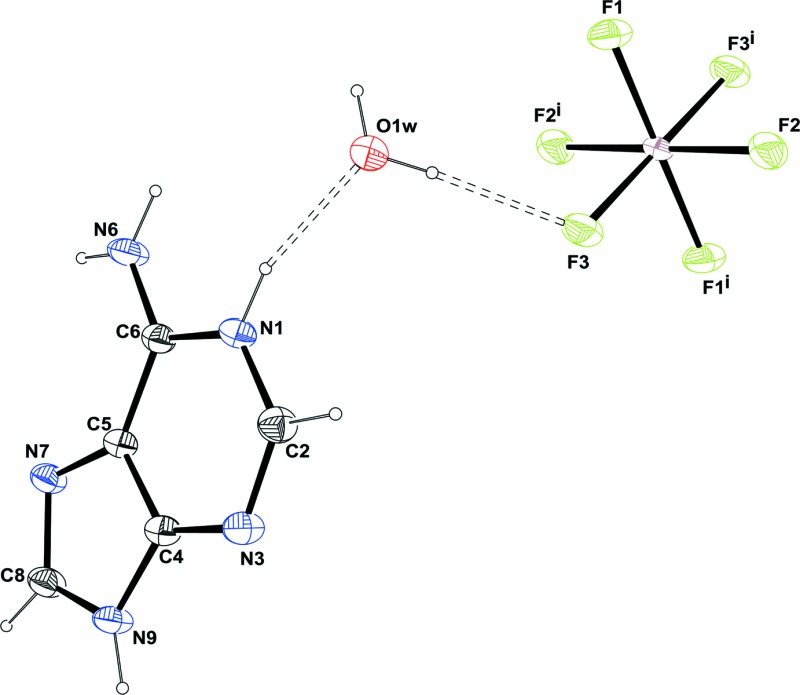
The principal structural units in the title compound. Displacement ellipsoids are drawn at the 30% probability level. H atoms are represented as small spheres of arbitrary radius. Hydrogen bonds are shown as dashed lines. [Symmetry code: (i) -x, -y, 2 - z]

**Fig. 2.**
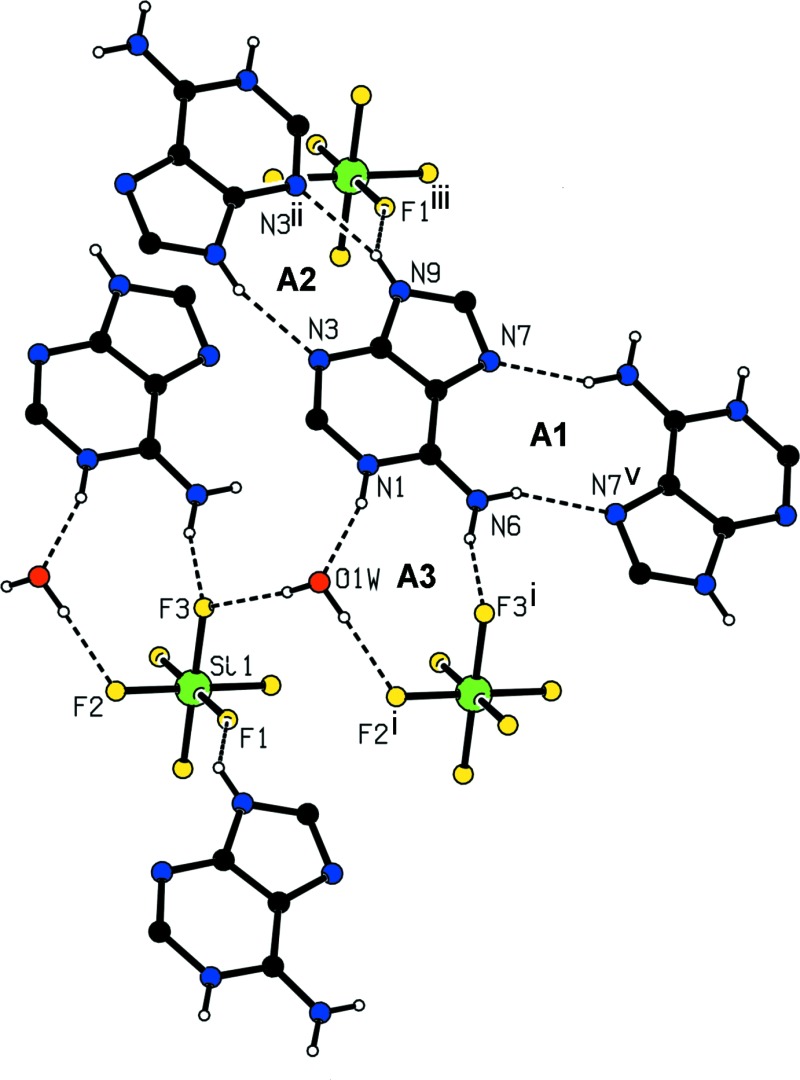
Partial packing view of the title compound, showing the formation of R22(10) (A1) and R22(8) (A2) rings through N—H···N, N—H···O, N—H···F and O—H···F hydrogen bonds. For the sake of clarity, H atoms not involved in hydrogen bonding have been omitted. [Symmetry codes: (i) -x, y - 1/2, -z + 1/2; (ii) -x, y + 1/2, -z + 1/2; (iii) x, y - 1, z; (iv) -x, -y, -z + 1.].

**Fig. 3.**
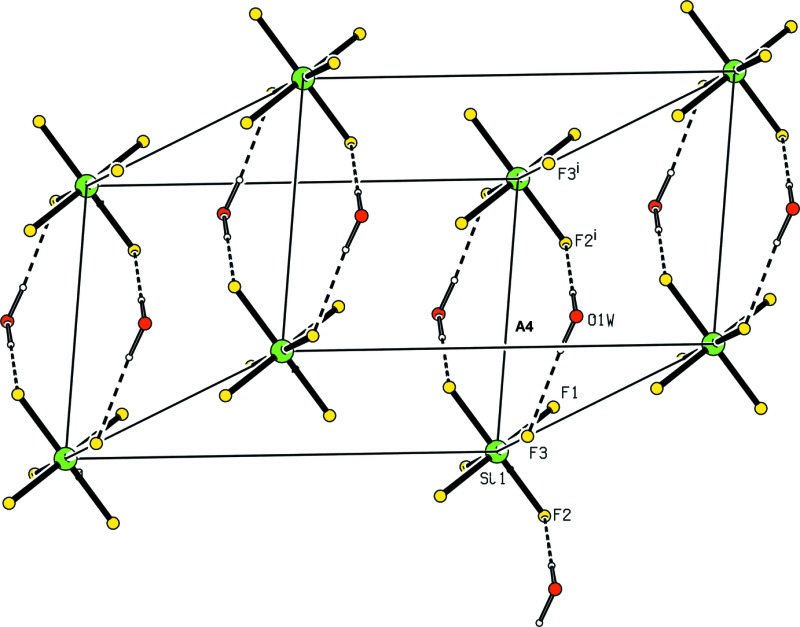
Partial packing view showing chains formed between water molecules and fluoridosilicate anions through O—H···F hydrogen bonds. For the sake of clarity, the cationic counterparts have been omitted. [Symmetry code: (i) x + 1, y, z]

Structure factors: contains datablock(s) I. DOI: 10.1107/S2056989014027005/wm5099Isup2.hkl

x y z . DOI: 10.1107/S2056989014027005/wm5099fig1.tif
The principal structural units in the title compound. Displacement ellipsoids are drawn at the 30% probability level. H atoms are represented as small spheres of arbitrary radius. Hydrogen bonds are shown as dashed lines. [Symmetry code: (i) −*x*, −*y*, 2 − *z*]

x y z x y z x y z x y z . DOI: 10.1107/S2056989014027005/wm5099fig2.tif
Partial packing view of the title compound, showing the formation of 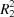
(10) (A1) and 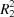
(8) (A2) rings through N—H⋯N, N—H⋯O, N—H⋯F and O—H⋯F hydrogen bonds. For the sake of clarity, H atoms not involved in hydrogen bonding have been omitted. [Symmetry codes: (i) −*x*, *y* − 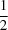, −*z* + 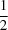; (ii) −*x*, *y* + 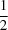, −*z* + 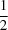; (iii) *x*, *y* − 1, *z*; (iv) −*x*, −*y*, −*z* + 1.].

x y z . DOI: 10.1107/S2056989014027005/wm5099fig3.tif
Partial packing view showing chains formed between water mol­ecules and fluorido­silicate anions through O—H⋯F hydrogen bonds. For the sake of clarity, the cationic counterparts have been omitted. [Symmetry code: (i) *x* + 1, *y*, *z*]

**Fig. 4.**
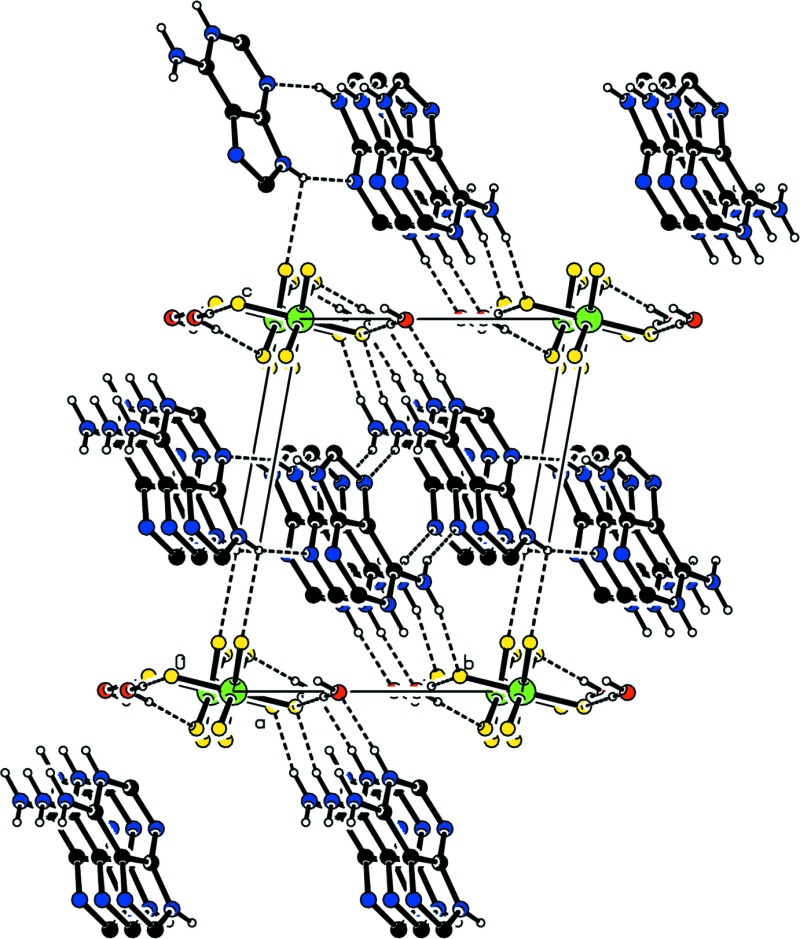
Packing view in a projection aproximately along [100] showing the formation of layers parallel to (001). Hydrogen bonds are shown as dashed lines. H atoms not involved in hydrogen bonding have been omitted for clarity.

. DOI: 10.1107/S2056989014027005/wm5099fig4.tif
Packing view in a projection aproximately along [100] showing the formation of layers parallel to (001). Hydrogen bonds are shown as dashed lines. H atoms not involved in hydrogen bonding have been omitted for clarity.

CCDC reference: 1038389

Additional supporting information:  crystallographic information; 3D view; checkCIF report

## Figures and Tables

**Table 1:** Hydrogen-bond geometry (, )

*D*H*A*	*D*H	H*A*	*D* *A*	*D*H*A*
O1*W*H1*W*F2^i^	0.85	1.88	2.7307(14)	178
O1*W*H2*W*F3	0.80	1.95	2.7553(14)	174
N1H1O1*W*	0.86	1.88	2.7059(15)	162
N9H9N3^ii^	0.86	2.13	2.9378(17)	157
N9H9F1^iii^	0.86	2.54	3.0009(14)	115
N6H6*A*F3^i^	0.86	1.98	2.7917(14)	157
N6H6*A*F1^iv^	0.86	2.61	3.2906(15)	137
N6H6*B*N7^v^	0.86	2.15	2.9648(18)	159

Symmetry codes: (i) 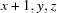; (ii) 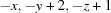; (iii) 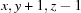; (iv) 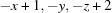; (v) 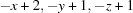.

## supplementary crystallographic information

### S1. Experimental

Crystals of the title compound were grown from aqueous solution by dissolving 1 mmol SiO_2_ and 2 mmol adenine in hydrofluoric acid (HF). The solutions were slowly evaporated to dryness for a couple of weeks. Some colourless crystals were isolated under a polarizing microscope for X-ray diffraction analysis.

### S2. Refinement

All H atoms attached to C or N atoms were fixed geometrically and treated as riding with C—H = 0.93 Å and N—H = 0.86 Å with *U*_iso_(H) = 1.2*U*_eq_(C or N). H atoms of the water molecule were located in difference Fourier maps and included in the subsequent refinement using restraints (O—H= 0.82 (1) Å and H···H = 1.38 (2) Å) with *U*_iso_(H) = 1.5*U*_eq_(O).

### Crystal data

**Table:**

2C_5_H_6_N_5_^+^·SiF_6_^2^^−^·2H_2_O	*Z* = 1
*M**_r_* = 450.42	*F*(000) = 230
Triclinic, *P*1	*D*_x_ = 1.762 Mg m^−^^3^
Hall symbol: -P 1	Mo *K*α radiation, λ = 0.71073 Å
*a* = 5.7500 (7) Å	Cell parameters from 2981 reflections
*b* = 7.8504 (3) Å	θ = 5.2–27.5°
*c* = 10.0884 (6) Å	µ = 0.24 mm^−^^1^
α = 79.141 (6)°	*T* = 295 K
β = 84.534 (17)°	Lath, colourless
γ = 71.774 (9)°	0.55 × 0.12 × 0.07 mm
*V* = 424.47 (6) Å^3^	

### Data collection

**Table:**

Nonius KappaCCD diffractometer	1757 reflections with *I* > 2σ(*I*)
Radiation source: fine-focus sealed tube	*R*_int_ = 0.018
Detector resolution: 9 pixels mm^-1^	θ_max_ = 27.5°, θ_min_ = 5.2°
CCD rotation images, thick slices scans	*h* = −6→7
4025 measured reflections	*k* = −10→10
1923 independent reflections	*l* = −13→13

### Refinement

**Table:**

Refinement on *F*^2^	Primary atom site location: structure-invariant direct methods
Least-squares matrix: full	Secondary atom site location: difference Fourier map
*R*[*F*^2^ > 2σ(*F*^2^)] = 0.030	Hydrogen site location: inferred from neighbouring sites
*wR*(*F*^2^) = 0.082	H-atom parameters constrained
*S* = 1.03	*w* = 1/[σ^2^(*F*_o_^2^) + (0.0373*P*)^2^ + 0.1468*P*] where *P* = (*F*_o_^2^ + 2*F*_c_^2^)/3
1923 reflections	(Δ/σ)_max_ = 0.001
133 parameters	Δρ_max_ = 0.28 e Å^−^^3^
0 restraints	Δρ_min_ = −0.22 e Å^−^^3^

### Special details

**Table:**

Geometry. All e.s.d.'s (except the e.s.d. in the dihedral angle between two l.s. planes) are estimated using the full covariance matrix. The cell e.s.d.'s are taken into account individually in the estimation of e.s.d.'s in distances, angles and torsion angles; correlations between e.s.d.'s in cell parameters are only used when they are defined by crystal symmetry. An approximate (isotropic) treatment of cell e.s.d.'s is used for estimating e.s.d.'s involving l.s. planes.
Refinement. Refinement of *F*^2^ against ALL reflections. The weighted *R*-factor *wR* and goodness of fit *S* are based on *F*^2^, conventional *R*-factors *R* are based on *F*, with *F* set to zero for negative *F*^2^. The threshold expression of *F*^2^ > σ(*F*^2^) is used only for calculating *R*-factors(gt) *etc*. and is not relevant to the choice of reflections for refinement. *R*-factors based on *F*^2^ are statistically about twice as large as those based on *F*, and *R*- factors based on ALL data will be even larger.

### Fractional atomic coordinates and isotropic or equivalent isotropic displacement parameters (Å^2^)

**Table:**

	*x*	*y*	*z*	*U*_iso_*/*U*_eq_	
Si1	0.0000	0.0000	1.0000	0.02519 (13)	
F1	0.16598 (16)	−0.01595 (12)	1.13081 (8)	0.0417 (2)	
F2	−0.25721 (16)	0.04669 (12)	1.09945 (9)	0.0432 (2)	
F3	−0.03226 (17)	0.22671 (11)	0.95904 (9)	0.0410 (2)	
O1W	0.36312 (19)	0.33709 (13)	0.99720 (11)	0.0411 (3)	
H1W	0.4831	0.2480	1.0279	0.062*	
H2W	0.2551	0.2970	0.9859	0.062*	
N1	0.4082 (2)	0.56497 (15)	0.76571 (10)	0.0304 (2)	
H1	0.4261	0.4920	0.8417	0.036*	
N7	0.7063 (2)	0.69851 (15)	0.44077 (11)	0.0322 (3)	
N9	0.3304 (2)	0.89656 (15)	0.41590 (11)	0.0339 (3)	
H9	0.2111	0.9840	0.3789	0.041*	
N6	0.8149 (2)	0.42421 (16)	0.70564 (12)	0.0384 (3)	
H6A	0.8298	0.3513	0.7817	0.046*	
H6B	0.9376	0.4154	0.6487	0.046*	
N3	0.1313 (2)	0.80786 (16)	0.63114 (11)	0.0344 (3)	
C6	0.6057 (2)	0.54850 (16)	0.67661 (12)	0.0277 (3)	
C4	0.3254 (2)	0.79502 (17)	0.54071 (12)	0.0287 (3)	
C5	0.5581 (2)	0.67381 (16)	0.55526 (12)	0.0268 (3)	
C8	0.5605 (3)	0.83344 (18)	0.36060 (14)	0.0347 (3)	
H8	0.6093	0.8810	0.2748	0.042*	
C2	0.1861 (3)	0.68935 (19)	0.74132 (13)	0.0334 (3)	
H2	0.0632	0.6901	0.8084	0.040*	

### Atomic displacement parameters (Å^2^)

**Table:**

	*U*^11^	*U*^22^	*U*^33^	*U*^12^	*U*^13^	*U*^23^
Si1	0.0258 (2)	0.0253 (2)	0.0204 (2)	−0.00695 (18)	−0.00429 (17)	0.00622 (16)
F1	0.0428 (5)	0.0461 (5)	0.0328 (4)	−0.0095 (4)	−0.0160 (4)	0.0021 (3)
F2	0.0341 (4)	0.0502 (5)	0.0354 (4)	−0.0076 (4)	0.0041 (3)	0.0054 (4)
F3	0.0494 (5)	0.0291 (4)	0.0412 (5)	−0.0126 (4)	−0.0109 (4)	0.0080 (3)
O1W	0.0376 (6)	0.0361 (5)	0.0458 (6)	−0.0123 (4)	−0.0092 (4)	0.0080 (4)
N1	0.0364 (6)	0.0310 (5)	0.0209 (5)	−0.0091 (4)	−0.0034 (4)	0.0016 (4)
N7	0.0338 (6)	0.0308 (5)	0.0266 (5)	−0.0067 (5)	−0.0001 (4)	0.0027 (4)
N9	0.0354 (6)	0.0298 (5)	0.0279 (6)	−0.0023 (5)	−0.0052 (4)	0.0050 (4)
N6	0.0354 (6)	0.0376 (6)	0.0297 (6)	−0.0022 (5)	−0.0020 (5)	0.0106 (5)
N3	0.0323 (6)	0.0355 (6)	0.0286 (6)	−0.0024 (5)	−0.0022 (4)	−0.0017 (4)
C6	0.0328 (6)	0.0260 (6)	0.0232 (6)	−0.0086 (5)	−0.0038 (5)	−0.0009 (4)
C4	0.0328 (7)	0.0265 (6)	0.0244 (6)	−0.0061 (5)	−0.0044 (5)	−0.0021 (4)
C5	0.0306 (6)	0.0247 (5)	0.0229 (6)	−0.0064 (5)	−0.0028 (5)	−0.0009 (4)
C8	0.0380 (7)	0.0332 (6)	0.0270 (6)	−0.0079 (5)	−0.0013 (5)	0.0049 (5)
C2	0.0348 (7)	0.0361 (7)	0.0271 (6)	−0.0081 (5)	0.0008 (5)	−0.0051 (5)

### Geometric parameters (Å, º)

**Table:**

Si1—F1	1.6646 (8)	N9—C4	1.3605 (16)
Si1—F1^i^	1.6646 (8)	N9—C8	1.3637 (19)
Si1—F2	1.6867 (9)	N9—H9	0.8600
Si1—F2^i^	1.6867 (9)	N6—C6	1.3080 (17)
Si1—F3	1.7041 (8)	N6—H6A	0.8600
Si1—F3^i^	1.7041 (8)	N6—H6B	0.8600
O1W—H1W	0.8476	N3—C2	1.3015 (17)
O1W—H2W	0.8046	N3—C4	1.3632 (17)
N1—C2	1.3543 (18)	C6—C5	1.4081 (16)
N1—C6	1.3681 (17)	C4—C5	1.3803 (18)
N1—H1	0.8600	C8—H8	0.9300
N7—C8	1.3154 (17)	C2—H2	0.9300
N7—C5	1.3892 (16)		
			
F1—Si1—F1^i^	180.000 (1)	C4—N9—H9	126.5
F1—Si1—F2	89.86 (5)	C8—N9—H9	126.5
F1^i^—Si1—F2	90.14 (5)	C6—N6—H6A	120.0
F1—Si1—F2^i^	90.14 (5)	C6—N6—H6B	120.0
F1^i^—Si1—F2^i^	89.86 (5)	H6A—N6—H6B	120.0
F2—Si1—F2^i^	180.0	C2—N3—C4	112.33 (12)
F1—Si1—F3	90.31 (4)	N6—C6—N1	120.97 (11)
F1^i^—Si1—F3	89.69 (4)	N6—C6—C5	125.23 (12)
F2—Si1—F3	89.68 (5)	N1—C6—C5	113.80 (11)
F2^i^—Si1—F3	90.32 (5)	N9—C4—N3	127.47 (12)
F1—Si1—F3^i^	89.69 (4)	N9—C4—C5	105.20 (11)
F1^i^—Si1—F3^i^	90.31 (4)	N3—C4—C5	127.32 (11)
F2—Si1—F3^i^	90.32 (5)	C4—C5—N7	111.11 (11)
F2^i^—Si1—F3^i^	89.68 (5)	C4—C5—C6	117.64 (12)
F3—Si1—F3^i^	180.000 (1)	N7—C5—C6	131.23 (12)
H1W—O1W—H2W	107.5	N7—C8—N9	113.25 (12)
C2—N1—C6	123.90 (11)	N7—C8—H8	123.4
C2—N1—H1	118.1	N9—C8—H8	123.4
C6—N1—H1	118.1	N3—C2—N1	125.00 (13)
C8—N7—C5	103.37 (11)	N3—C2—H2	117.5
C4—N9—C8	107.06 (11)	N1—C2—H2	117.5

Symmetry code: (i) −*x*, −*y*, −*z*+2.

### Hydrogen-bond geometry (Å, º)

**Table:**

*D*—H···*A*	*D*—H	H···*A*	*D*···*A*	*D*—H···*A*
O1*W*—H1*W*···F2^ii^	0.85	1.88	2.7307 (14)	178
O1*W*—H2*W*···F3	0.80	1.95	2.7553 (14)	174
N1—H1···O1*W*	0.86	1.88	2.7059 (15)	162
N9—H9···N3^iii^	0.86	2.13	2.9378 (17)	157
N9—H9···F1^iv^	0.86	2.54	3.0009 (14)	115
N6—H6*A*···F3^ii^	0.86	1.98	2.7917 (14)	157
N6—H6*A*···F1^v^	0.86	2.61	3.2906 (15)	137
N6—H6*B*···N7^vi^	0.86	2.15	2.9648 (18)	159

Symmetry codes: (ii) *x*+1, *y*, *z*; (iii) −*x*, −*y*+2, −*z*+1; (iv) *x*, *y*+1, *z*−1; (v) −*x*+1, −*y*, −*z*+2; (vi) −*x*+2, −*y*+1, −*z*+1.

## References

[bb1] Belhouas, R., Bouacida, S., Boudaren, C., Daran, J.-C. & Roisnel, T. (2012). *Acta Cryst.* E**68**, o1791–o1792. 10.1107/S1600536812021587 PMC3379370 22719568

[bb3] Bouacida, S., Merazig, H., Beghidja, A. & Beghidja, C. (2005*a*). *Acta Cryst.* E**61**, m1153–m1155.

[bb4] Bouacida, S., Merazig, H., Beghidja, A. & Beghidja, C. (2005*b*). *Acta Cryst.* E**61**, m2072–m2074.

[bb5] Bouacida, S., Merazig, H., Beghidja, A. & Beghidja, C. (2005*c*). *Acta Cryst.* E**61**, m577–m579.

[bb6] Bouacida, S., Merazig, H. & Benard-Rocherulle, P. (2006). *Acta Cryst.* E**62**, o838–o840.

[bb7] Burla, M. C., Caliandro, R., Camalli, M., Carrozzini, B., Cascarano, G. L., De Caro, L., Giacovazzo, C., Polidori, G. & Spagna, R. (2005). *J. Appl. Cryst.* **38**, 381–388.

[bb8] Burnett, M. N. & Johnson, C. K. (1996). *ORTEPIII*. Report ORNL-6895. Oak Ridge National Laboratory, Tennessee, USA.

[bb9] Duisenberg, A. J. M., Kroon-Batenburg, L. M. J. & Schreurs, A. M. M. (2003). *J. Appl. Cryst.* **36**, 220–229.

[bb11] Farrugia, L. J. (2012). *J. Appl. Cryst.* **45**, 849–854.

[bb12] Janiak, C. (2000). *J. Chem. Soc. Dalton Trans.* pp. 3885–3896.

[bb13] Otwinowski, Z. & Minor, W. (1997). *Methods in Enzymology*, Vol. 276, *Macromolecular Crystallography*, Part A, edited by C. W. Carter Jr & R. M. Sweet, pp. 307–326. New York: Academic Press.

[bb14] Sheldrick, G. M. (2008). *Acta Cryst.* A**64**, 112–122. 10.1107/S0108767307043930 18156677

[bb15] Spek, A. L. (2009). *Acta Cryst.* D**65**, 148–155. 10.1107/S090744490804362X PMC2631630 19171970

