# Supplementary figures and images for: Crystal structure of bis­(9H-6-amino­purin-1-ium) hexa­fluorido­silicate(IV) dihydrate
Source: Acta Crystallogr E Crystallogr Commun. 2015 Jan 3;71(Pt 2):o72–3. doi: 10.1107/S2056989014027005 (PMC4384624; doi:10.1107/S2056989014027005)

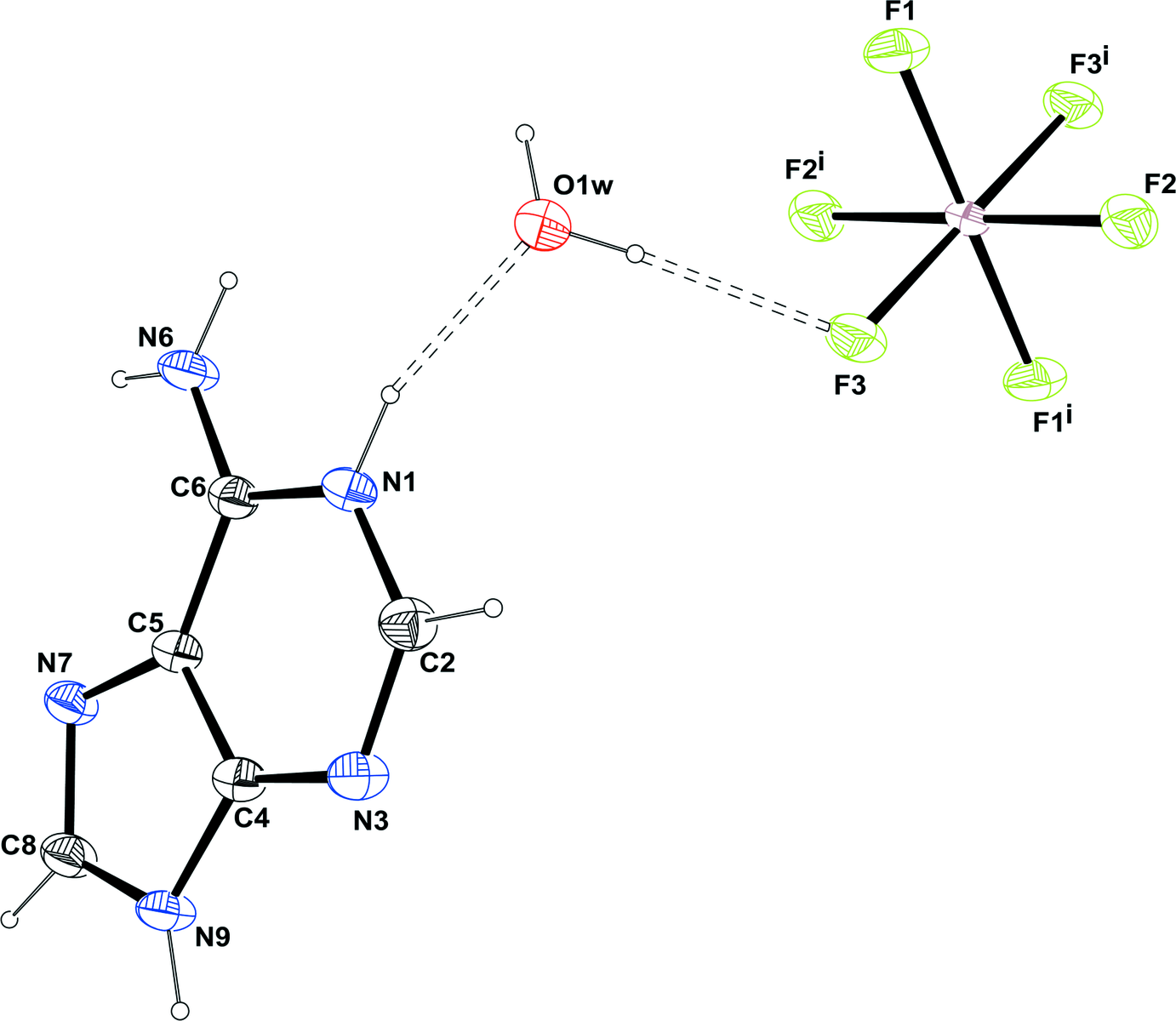

Supplement: Supplementary file 3 [file e-71-00o72-fig1.tif]

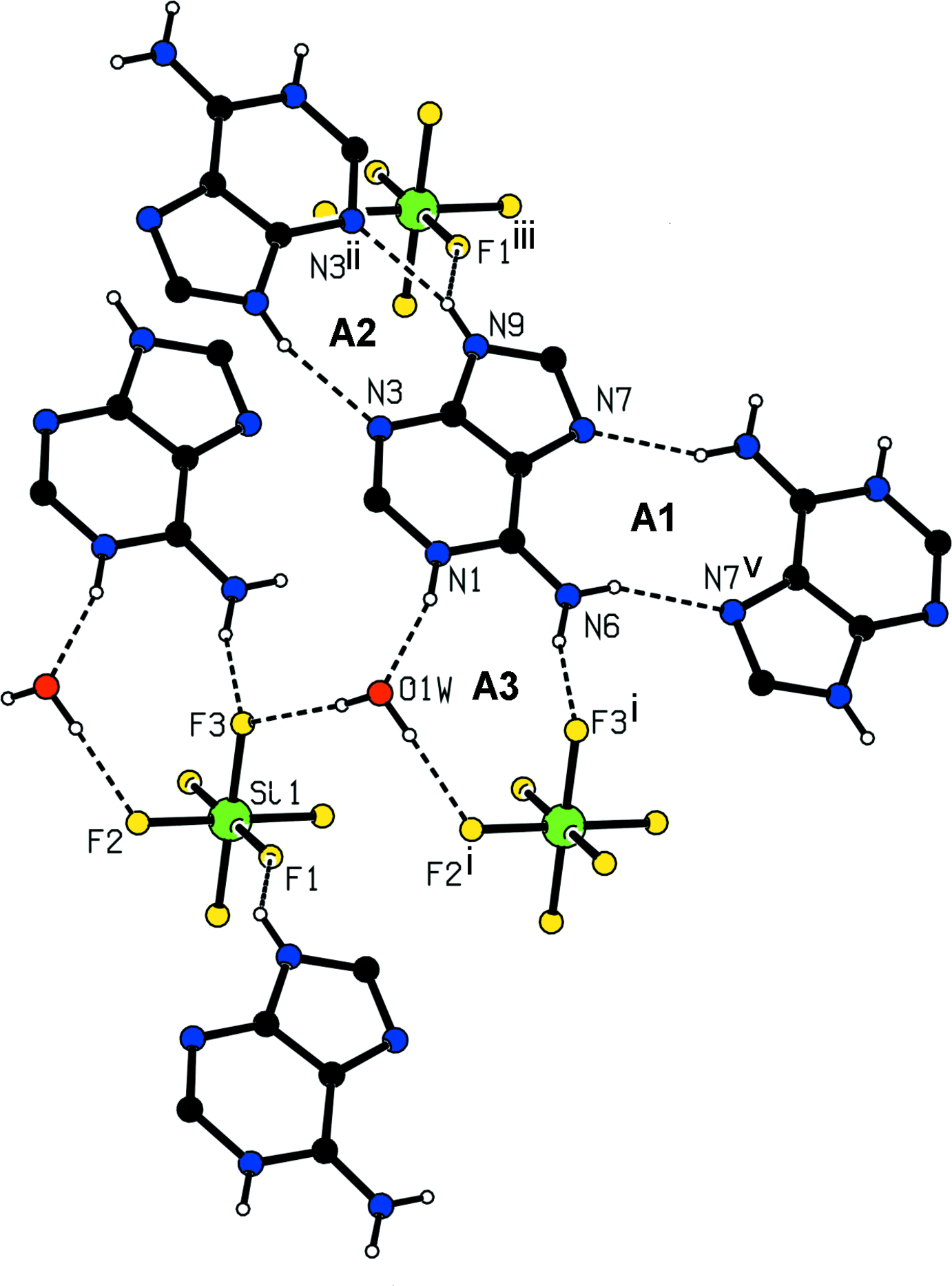

Supplement: Supplementary file 4 [file e-71-00o72-fig2.tif]

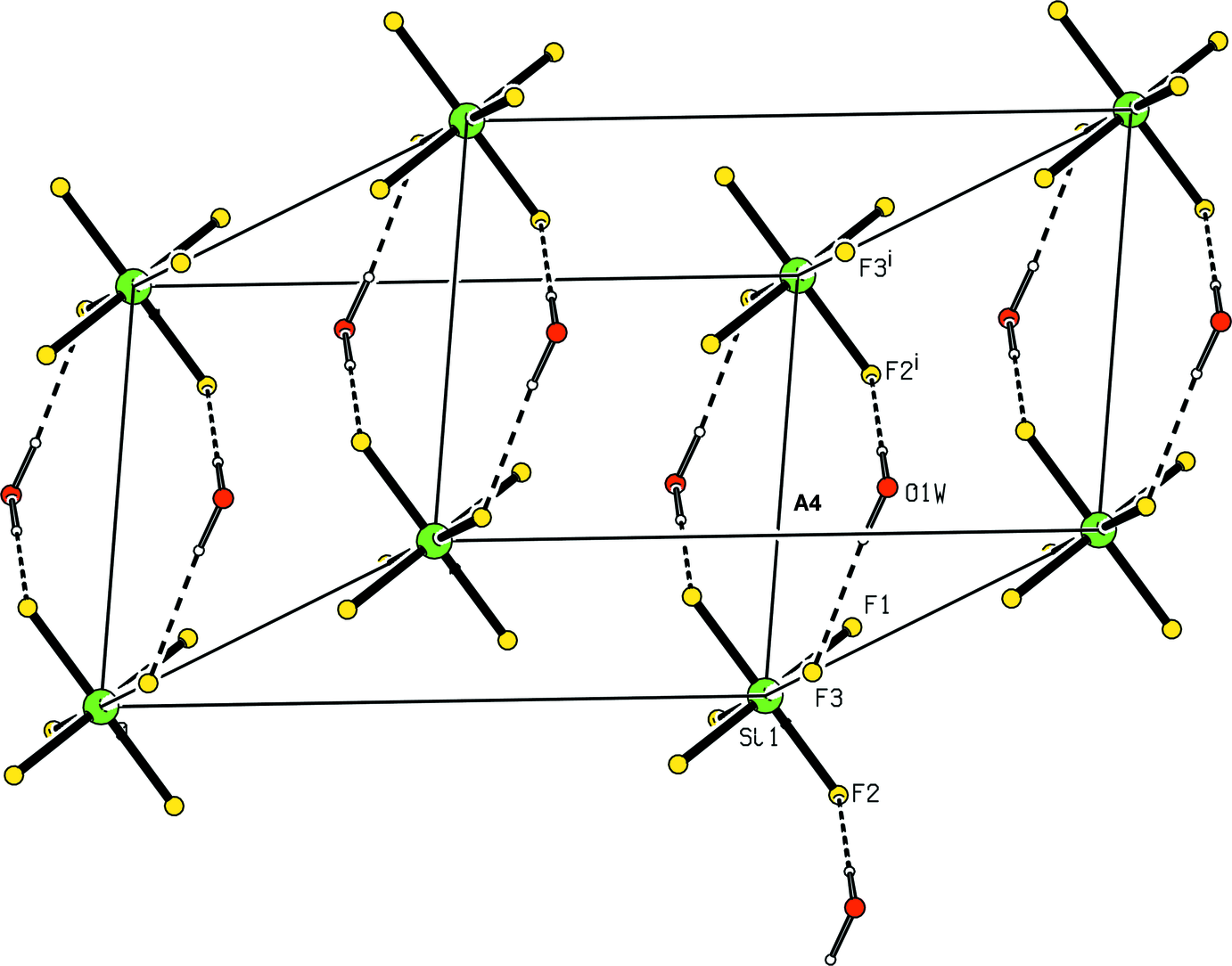

Supplement: Supplementary file 5 [file e-71-00o72-fig3.tif]

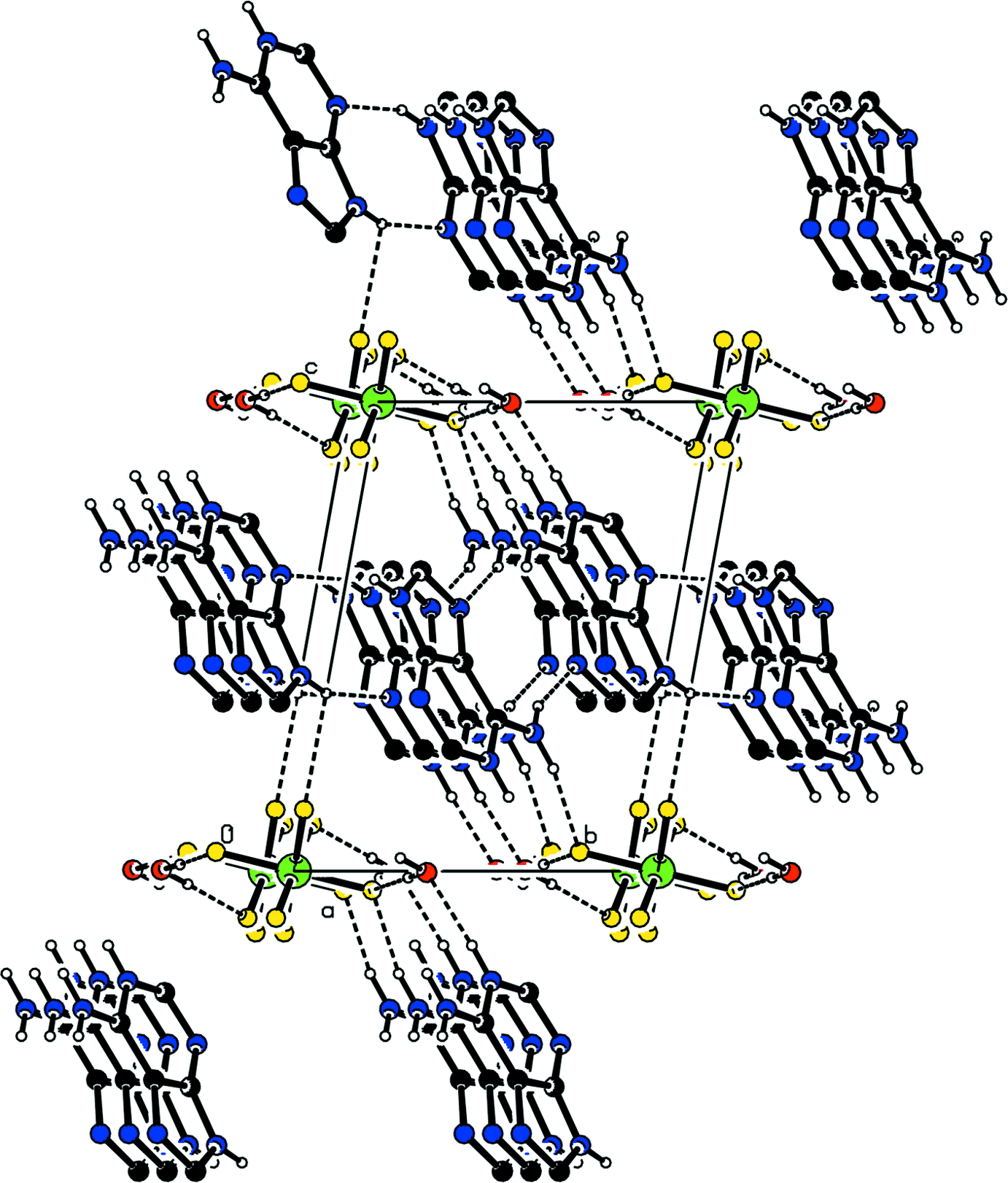

Supplement: Supplementary file 6 [file e-71-00o72-fig4.tif]
